# Autoinfection as a Factor in Diseases of Infants and Children1Read at the thirty-third annual meeting of the Medical Association of Central New York, held at Rochester, N. Y., October 16, 1900.

**Published:** 1900-12

**Authors:** J. Roberts Johnson

**Affiliations:** Syracuse, N. Y.


### BUFFALO MEDICAL JOURNAL.

VOL. XL.—LVI. DECEMBER, 1900. NO. 5

### Original Communications.

### AUTOINFECTION AS A FACTOR IN DISEASES OF INFANTS AND CHILDREN.[1]

BY J. ROBERTS JOHNSON, M. D., Syracuse, N. Y.

THAT which we call the "germ theory" of disease was broadly hinted at many years before the dawn of the Christian era. Nearly two thousand years ago a certain philosopher said: "It is also to be noticed if there be any marshy places that certain minute animals breed there which are invisible to the eye, and yet getting into the system through mouth and nostrils cause serious disorders." Does it not seem remarkable that an idea which should become a fundamental principle of pathology and mark a new era in scientific medicine should be taught centuries ago?

Many of the conclusions of the early investigators have proven false, and it was not until the microscope revealed the hitherto unseen that much of definite knowledge has been reached and practical results demonstrated. Many of the etiological factors of disease were until now matters of theoretical speculation, and upon this ground therapy was based.

The microscope has disclosed in not a few instances the cause of disease and therapeutically we deal with definite and known organisms. The study of the specific bacteria as an origin of disease has opened up a new an ever increasing field of scientific research, and one which in the last two decades has done as much for rational medicine as all else combined. With each new finding in pathology, naturally comes the question does it aid in curing disease? Heretofore the bacteriologist has been compelled to admit that the demonstra-

1. Read at the thirty-third annual meeting of the Medical Association of Central New York, held at Rochester, N. Y., October 16, 1900.

tion of the specific cause in not a few infectious diseases which has been obtained through his efforts, has not resulted in the discovery of the specific treatment of these diseases. This, in relation to some morbid conditions at least, can no longer be said.

Self-infection is the infection of an organism by a ferment or poison generated within itself. The clinical picture developed depends upon the nature and virulence of the poison, the susceptibility of the patient, and the inherent power of the subject in combating disease. Starr, in relation to convulsions, says:

An infant is a bundle of nerves and nerve centers, and reflexes, in a state of great activity, prepared to receive, store up, and reënergise a world full of new impressions suddenly thrust upon it. While the nervous system of the adult has acquired the steadiness of long habit and has but to repair waste, that of the infant has all of the delicacy and instability of newly formed and highly impressionable protoplasm and besides having to preside over the processes of repair, it must govern the growth of the whole organism.

What is said of convulsive seizures is equally true of the causes of autoinfection in infants and children, which differs from that of the adult by its more frequent occurrence, rapidity of onset and more profound degree of expression. Cell life in the infant is very active and its assimilative powers are equally energetic; any change in the metabolic processes soon produce a morbid state. The infant may be infected by poisonous germs and toxic substances, which are only recognised when some change of the normal functions takes place and more or less rapidly disease phenomena appears.

The important rôle of autoinfection in diseases of infants and children is becoming better understood, and explains the increased dangers of mixed infections which it adds to that of the diseases which are purely infectious, contagious, or both, and which of course, come from exposure to conditions wholly foreign to the organism.

That bacteria infest the system continuously is well recognised and that, in a normal condition we are, in a measure, immune to their effects. The infant by reason of its rapid cell proliferation, its natural and often unnatural diet, its surroundings, and greater susceptibility to autoinfection succumbs to disease much more readily than does the adult. Childhood is immune to few diseases of bacterial origin.

In the elaboration of bacteria in the system, a toxin is developed which produces a series of clinical circumstances, leading to dangerous or even fatal results. The early recognition of this truth is important if the poison is to be annulled.

With our present knowledge, it is impossible to state whether certain morbid conditions are due in their entirety to defective metabolism or the result of bacterial toxins or both combined. That there is no sharp distinction between the pathogenic and nonpathogenic bacteria is recognised. Under certain conditions, the virulence of disease bacteria, is annulled, while the most innocent may become virulent.

Views as to the toxicity of certain secretions within the animal economy are in an unsettled condition. The trend of medical thought, however, is to regard them as germicidal in character, so that each is ready to guard the entrance of poisonous material into the system, or if admitted to destroy its pernicious effects.

When we consider the enormous mortality-rate in infants, and to what an extent autoinfection is an etiological factor in producing such appalling records, it stands us well in hand to early recognise such infection, that not alone the therapeutic measures of long recognised value may be used, but also call to our aid the latest and best which organo- and serum therapy offers.

The sources of autoinfection begin to operate in the early period of fetal life through the placental circulation, and may remain latent or develop into the complete cycle of disease factors and even death "in utero." Deep inspirations of the infant during the last stage of labor may carry infected material into the bronchial tubes, producing a broncho-pneumonia, which soon proves fatal.

In the early days of infantile life, pyogenic germs may enter the system, the most frequent avenue being the umbilical wound. The clinical picture varies according to the character and location of the inflammation. The severity of the symptoms depends somewhat on the nature of the bacteria, which excites the disease and of their virulence at the time of their infection.

A meningitis, a pneumonia, joint suppuration or an erysipelas may ensue according to the area involved. Since antisepsis has receivd its due recognition, these diseases are fortunately less often seen.

The gastro-intestinal route normally is tenanted by germs virulent and benign. In health, the infant suffers no harm from their presence, but if the natural resistance is lowered, either because food of improper quality or quantity is allowed, then bacterial fermentation takes place and discomfort and suffering attend. Poisonous products are rapidly formed within the system affecting not alone the gastro-intestinal route, but the entire economy.

Not infrequently the nervous centers are profoundly influenced and convulsions or meningeal complications follow: some of the symptoms in these severe cases point to a septic condition and to aid nature in its eliminative efforts is clearly indicated.

Pure milk, which is conceded to be the "staff of life" for the infant in health, may become under certain circumstances pabulum for the proliferation of various disease producing organisms in the intestinal route, and, by rapid growth, soon present the clinical picture of that prevalent and dangerous malady which modern writers have properly named acute milk infection. By the withdrawal of milk from the infant during the early stage of the disease supplemented by other remedial measures indicated, far better results are now obtained than was considered possible a few years ago.

The infective bacteria chiefly inhabit the lower intestinal tract. By means of lavage, purgatives, and colonic irrigations much of the products upon which bacteria thrive and multiply is gotten rid of, and hordes of deadly organisms removed. Such a procedure is rational and easy of application. Much has been learned not alone from the grosser examination of the infant stools, but by the microscopic as well.

All infant stools contain bacteria, but the prognostic value is very great, if we know that the streptococci are absent. The treatment is less difficult in the simple coli varieties. Such a distinction is quite imperative today, if the infective nature of the disease is considered, not alone as to the child already sick, but that others of the same family or institution may not be infected. In this country nearly one half of all deaths below one year are caused by gastro-intestinal diseases. Outside of this class the entire infectious group cannot claim an equal mortality. It is well established that children of this class do badly if huddled together; mild cases become severe, and previously healthy children are made sick. The same vigilance is necessary as in other infectious diseases. Isolation and perfect cleanliness should be observed. Prophylaxis is of paramount value. If the child is artificially fed, let the food be as clean, correctly prepared, and given as our latest knowledge directs. The milk supply is of chief value, not alone as to its bacterial condition, but as to grosser impurities. If autoinfection is to be prevented, it must largely be by the giving of food free from autotoxic substances and bacterial toxins.

Post mortem examination reveals that in a proportion of cases microbes are found in the intestinal mucosa, principally in the lower portion, the duodenum and jejunum being quite free. In the mild gastro-enteritis of babies, the lesions are chiefly due to the action of chemical substances taken in with the food, or elaborated within the digestive tube. The lesions so produced, serve as points of entrance for bacteria, and these in turn cause further morbid changes or even septicemia. If the problem of keeping the digestive route constantly in a normal condition were solved, some of the reflex phenomena whose etiology is not entirely clear would fail to appear.

The mouth and naso-pharynx are fertile fields for autoinfection, because in these cavities various bacteria are omnipresent, and any abrasion of the lining membranes, or a lowering of their tone and resisting power may favor their entrance, proliferation and consequent self-infection. In this connection, I wish to quote from a recent article by Dr. Francis Huber:

> A normal mucous membrane is the best safeguard against the onset of a number of infectious microörganisms. The invasion of diphtheria, tuberculosis, and now and then meningitis is favored by an abnormal condition of the nasal and pharyngeal mucous membrane. The best preventive therefore is to keep the mucous membrane in a healthy state. The eloquent appeals in favor of a daily naso-pharyngeal toilet have added somewhat in popularising this method. Anatomists have clearly demonstrated the direct lymphatic communication between the vessels in the naso-pharyngeal mucous membrane and those at the base of the brain. Bacteriologists have reported the presence of microörganisms in the nose and throat similar to those found in many cases of meningitis. Clinical observations show that the different varieties of meningitis are most commonly observed between the ages of three and five years, at a time when naso-pharyngeal disturbances are very common. The intimate lymphatic connection referred to, and the identity of the microorganisms in the naso-pharynx and those found in a large number of cases of meningitis tend to explain the etiology of many heretofore obscure inflammations of the brain and meninges.

The absorptive power of the naso-pharyngeal membrane is marked. Microörganisms rapidly multiply in these unhealthy secretions and tissues, and if undisturbed may be taken by the blood current to be a nidus of disease in some other part of the body. The naso-pharynx of every child suffering from infectious disease should daily be douched with the physiological salt solution or some mild alkaline antiseptic mixture. As a prophylactic measure, this should be done in all children exposed to diphtheria or scarlet fever, and the like.

Lymphoid tissue in the young is prone to take on morbid change. It readily appropriates pathogenic germs and will give up its own entity by calcific degeneration or suppuration rather than inoculate the system. Tuberculous adentitis is but the manifestation of infection received directly from the pharynx, or from hypertrophied tonsils and adenoid vegetations, these latter strongly favor tuberculosis and should early receive surgical treatment. The lymph nodes of the neck and thorax are frequently the earliest seat of tuberculous deposits and in many cases they are foci from which, secondary infection of the lungs, brain and joints may occur.

Some of the meningeal inflammations in children, in which the etiology is obscure, are doubtless of infective origin. Lumbar puncture as a diagnostic measure often reveals the streptococci and pneumococci in the simple cerebral or cerebro-spinal types, while the tubercle bacilli is most frequently found in the tubercular variety.

From post mortem findings in the wall of the intestines, some cases of tubercular meningitis are now considered to be a secondary infection. Autopsy does not always reveal in these tubercular cases sufficient morbid change to cause death. Is it not possible that other infection, more deadly, is a complicating factor?

That the pneumococcus is an etiological factor in the pneumonias of children is generally admitted. In some of these cases the local expression is not at all proportionate to the violence of the general symptoms at least in the early stages. In such conditions there is probably a mixed infection in which the streptococci played a deadly part. This organism is much more destructive than is the pneumococci, or even the tubercle bacilli. In these severe cases, especially of a mixed type, recent clinical reports favor the use of antistreptococcic serum, as an agent of value; other medication is not to be ignored. A broncho-pneumonia accompanying pertussis, which so often proves fatal, or that which attends the latter stages of scarlet fever or diphtheria is usually a secondary streptococcic infection.

A toxic condition of the system arising from delayed elimination of fecal matter, which may be reabsorbed by the intestine, is an important etiological factor in the functional neuroses of children; chorea, tetany, night terrors and dreaming, nocturnal enuresis, migraine and allied conditions and epilepsy belonging to this class. These diseases mostly occur at a time when overeating or eating of food unsuitable to the child's individual capacity is indulged in with resultant putrefaction or delayed elimination. On this premise the treatment of these diseases is largely dietetic. If indican in considerable quantity is found in the urine, it is a positive chemical test that autointoxication from the intestinal canal is in progress.

Constipated and dyspeptic children are apt to suffer from erythema, resulting from intestinal autoinfection, sometimes to such an extent that the diagnosis between it and scarlatina for a time is doubtful.

In the congenital absence of the thyroid gland, or of its perverted function when present, a picture of an acute or chronic poisoning is presented. This gland has an antitoxic function and thereby prevents infection of the organism by destroying or changing certain products of metabolism which would otherwise prove poisonous. Heretofore all attempts or remedial measures for the relief of this condition have failed because the class of remedies used empirically did not fulfil the indications as now understood. The brilliant success already achieved with thyroid medication in combating this autoinfection is a distinct advance in applied therapeutics.

Uric acid, sugar and other unresolved products of the economy whose elimination is mainly carried on through the kidneys, so irritate their epithelial lining that inflammation and abrasion of the surface facilitate the entrance and activity of toxin-producing bacteria and their consequent absorption in the system, disturbs the nerve centers and causes much of the ill-defined malaise, and listlessness which are oftentimes attributed to idiosyncracies of temperament only. The examination of the urine chemical and microscopical gives data of much value. How frequently the source of severe headaches, migrating neuralgia, or of persistent vomiting may be reached by the examination of the urine in the form of uric acid and its attending power for autoinfection.

While admitting that positive conclusions regarding the causation of some diseased conditions is not conclusively settled, yet scientific investigation is rapidly unfolding the mystery of obscure etiology and placing therapeutics on a more rational basis.

In a word, prophylaxis is the means by which the impressionable infant organism is saved from the varied phenomena of autoinfection.

---

Unequal Width of the Pupils of the Insane.—Able (*Ungar. med. Presse*) investigated 326 patients with regard to the pupillary state. In sixty cases of progressive paralysis pupillary inequality (anisocoria) was present in 52 per cent.; in insanity of the congenital type, 27 per cent.; in epileptics, 26 per cent.; and in senile dementia, 25 per cent. As anisocoria is always a pathological phenomenon, these figures are significant.

